# Adenosine A_2A_ Receptor Blockade Ameliorates Mania Like Symptoms in Rats: Signaling to PKC-α and Akt/GSK-3β/β-Catenin

**DOI:** 10.1007/s12035-022-02977-2

**Published:** 2022-08-09

**Authors:** Heba Nasr Shalaby, Hala Fahmy Zaki, Afaf Abd Almonim Ain-Shoka, Reham Atef Mohammed

**Affiliations:** Department of Pharmacology and Toxicology, Faculty of Pharmacy, Cairo University, Cairo, Egypt

**Keywords:** Adenosine, Mania, Prefrontal cortex, PKC-α, GSK-3β, SNAP-25

## Abstract

Adenosinergic system dysfunction is implicated in the pathophysiology of multiple neuropsychiatric disorders including mania and bipolar diseases. The established synergistic interaction between A_2A_ and D_2_ receptors in the prefrontal cortex could highlight the idea of A_2A_ receptor antagonism as a possible anti-manic strategy. Hence, the present study was performed to examine the effect of a selective adenosine A_2A_ receptor blocker (SCH58261) on methylphenidate-induced mania-like behavior while investigating the underlying mechanisms. Rats were injected with methylphenidate (5 mg/kg/day, i.p.) for 3 weeks with or without administration of either SCH58261 (0.01 mg/kg/day, i.p.) or lithium (150 mg/kg/day, i.p.) starting from day 9. In the diseased rats, adenosine A_2A_R antagonism reduced locomotor hyperactivity and risk-taking behavior along with decreased dopamine and glutamate levels. Meanwhile, SCH58261 restored NMDA receptor function, suppressed PKC-α expression, down-regulated β-Arrestin-2, up-regulated *p*S473-Akt and *p*S9-GSK-3β. Further, SCH58261 promoted synaptic plasticity markers through increasing BDNF levels along with down-regulating GAP-43 and SNAP-25. The A_2A_ antagonist also reduced NF-κBp65 and TNF-α together with elevating IL-27 level giving an anti-inflammatory effect. In conclusion, suppression of PKC-α and modulation of Akt/GSK-3β/β-catenin axis through A_2A_R inhibition, could introduce adenosine A_2A_R as a possible therapeutic target for treatment of mania-like behavior. This notion is supported by the ability of the A_2A_R antagonist (SCH58261) to produce comparable results to those observed with the standard anti-manic drug (Lithium).

## Introduction

Adenosine is an endogenous neuromodulator involved in the regulation of multiple CNS functions. Adenosine acts at four receptor subtypes namely A_1_, A_2A_, A_2B_, and A_3_ [1]. Adenosine A_2A_ receptors (A_2A_R) are mainly expressed in the striatum and prefrontal cortex (PFC) where they interact with dopamine D_2_ receptors in both areas in antagonistic and synergistic manners, respectively [2]. Adenosinergic system dysfunction is implicated in the pathophysiology of several diseases [3]. Out of the four subtypes, A_2A_R are specifically appealing targets to control several mood disorders [4]. This could be related to adenosinergic modulation of glutamate and dopamine (DA), the two main key players for the proper functioning of both the ventral striatum and PFC [2]**.** Evidence suggests that schizophrenia induces upregulation of A_2A_R which could be a compensatory response to the reduced adenosine activity following such insults [5]**.** Upregulation of A_2A_R in turn leads to vicious cycles of excitotoxicity, inflammation and apoptosis raising the question if A_2A_ receptor blockade could possibly mitigate the brain damage caused by such ailments. Moreover, A_2A_R antagonists were proven to be neuroprotective in models of brain ischemia, Parkinson’s, multiple sclerosis and Huntington’s disease [6]**.** However, the role of A_2A_R blockade in manic disorders is still unclear.

The psychostimulant methylphenidate (MPH) that is widely prescribed for the treatment of attention deficit hyperactivity disorder (ADHD) [7] is also a well-accepted manic inducer [8]. It was reported that systemic injection of MPH is associated with behavioral sensitization evidenced by locomotor hyperactivity resembling those observed in mania [8]. Hence, in the current study, MPH was used as an inducer to mania-like symptoms in rats. Among other brain areas, PFC is a well-known target for MPH [9]**.** Under tight control of optimal dopaminergic tansmission, PFC regulates locomotor and behavior responses via connection to sensory and motor cortices [10]. In particular, MPH through eliciting dopaminergic hyperactivity interferes with PFC function and induces mania-like behavior [11]**.**

Increasing attention has been directed towards Protein kinase C*-α* (PKC-α) as a key enzyme which phosphorylates a variety of substrates and plays a pivotal role in different signaling cascades [12]. PKC-α is highly expressed in multiple brain regions that are involved in mood regulation, including PFC, hippocampus and amygdala [13]. In response to persistent activation of PKC-α, multiple targets such as Akt/GSK-3β/β-catenin axis, Growth-associated protein 43 (GAP-43), and Synaptosomal Associated Protein-25 (SNAP-25) are dysregulated [14]. Because of PKC-α’s important function in regulating essential activities, it’s believed that diminishing PKC-α activity might help in alleviating mania-like symptoms [15]. Interestingly, Akt (protein kinase B) promotes neuronal survival and synaptic plasticity through phosphorylating and hence inactivating downstream targets such as glycogen synthase kinase-3β (GSK-3β) [16]. As a consequence of GSK-3β inactivation, β-catenin is accumulated in the nucleus where it activates gene transcription and supports cell survival [17].

Synaptic plasticity is under tight control of the balance between excitatory and inhibitory signals where its deregulation is strongly associated with neuropsychiatric disorders [18]. This could be explained by altered expression of certain molecules such as GAP-43, SNAP-25 and brain-derived neurotrophic factor (BDNF). GAP-43 expression is known to be disrupted in bipolar disorder [19]. Indeed, overexpression of GAP-43 is suggestive for dysfunction in synaptic connections in autism and schizophrenia [20]. Notably, SNAP-25 is part of the soluble *N*-ethylmaleimide-sensitive factor attachment protein receptor (SNARE) protein complex. Overexpression of SNAP-25 in bipolar disorder was proven to impair synaptic plasticity and neurotransmission [21]. Among the other neurotrophins, BDNF is widely distributed in the cerebral cortex and hippocampus, where it plays a fundamental role in modulating neuronal survival and plasticity [22].

To this end, and to endorse the involvement of adenosine signaling cascade in the pathogenesis of mania, SCH58261, a selective adenosine A_2A_R antagonist, was used as a tool to investigate the impact of adenosine A_2A_R blockade on mania-like behavior-induced by MPH administration. This was accomplished via a series of behavioral and biochemical assessments, besides focusing on the PKC-α and its relation to the BDNF/Akt/GSK-3β/β-catenin trajectory in the rat PFC. To support the potential therapeutic role of A_2A_R blockade in this ailment, the effect of SCH58261 was compared to those of the established anti-manic drug (lithium).

## Materials and Methods

### Animals

Adult male Wistar rats (250–300 g) were obtained from the animal facility of the Faculty of Pharmacy, Cairo University (Cairo, Egypt). Rats were subjected to controlled environment at a constant temperature (25 ± 2 °C), humidity (60 ± 10% humidity), and light/dark (12/12 h) cycle. Standard chow diet and water were allowed during the experiment. Animal handling and experimental protocols were approved by the Research Ethics Committee of the Faculty of Pharmacy, Cairo University (Cairo, Egypt) (2547) and concurs with the US National Institutes of Health guide for the Care and Use of Laboratory Animals. All efforts were done to minimize the number of animals used.

### Drugs and Chemicals

Methylphenidate (MPH), SCH58261 and Lithium (Li) were purchased from Sigma-Aldrich Co. (St Louis, MO, USA). Both MPH and Li were freshly prepared daily in normal saline (0.9% w/v) in a volume of 2 ml/kg body weight, while SCH58261 was dissolved in DMSO in saline (1:3). All drugs were intraperitoneally injected. Chemicals were of the highest purity and analytical grade.

### Experimental Design

**Fig. 1:**
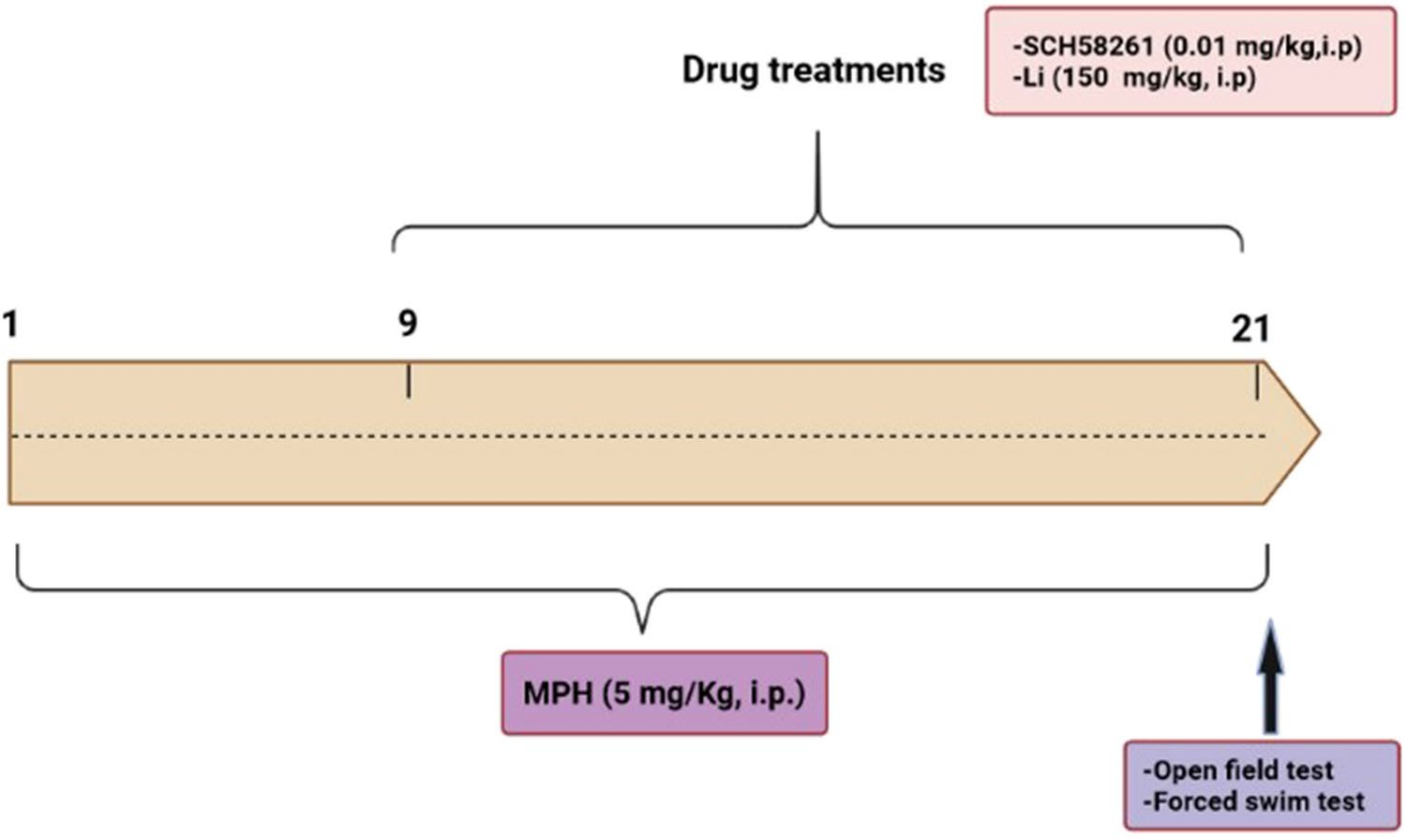
Schematic illustration of the experimental timeline. MPH: methylphenidate, Li: lithium

In the current study, 90 rats were randomly allocated into six groups (15 rats each). **Group 1** served as control where rats were injected DMSO in saline (1:3). **Group 2** served as SCH58261 control group where rats were administered SCH58261 (0.01 mg/kg, i.p.) [23] for 21 consecutive days**. Group 3** served as Li control group where rats were injected lithium (150 mg/kg, i.p.) [24] for 21 consecutive days. **The remaining rats** (n = 45) received a daily injection of MPH (5 mg/kg, i.p.) [25, 26] and recorded as diseased rats. Continuous injection of MPH altered behavioral activity as displayed in the open field (OFT) and forced swim (FST) tests on the 9^th^ day. Thereafter, MPH-treated rats were divided into 3 subgroups (15 rats each) and treated as follows: **group 4**: rats continued MPH injection (5 mg/kg, i.p.) for a total of 21 consecutive days and considered as MPH group. **Group 5**: rats received a daily injection of SCH58261 (0.01 mg/kg, i.p.) starting from the 9th day simultaneously with MPH injection. **Group 6**: rats received a daily injection of Li (150 mg/kg, i.p.) starting from the 9th day simultaneously with MPH injection [24] (Fig. 1). The duration of MPH injection was based on a previous study by Tamilselvan et al. [8] who reported that repeated administration of MPH-produced manic-like symptoms in mice and was confirmed by our pilot study (data not shown).

### Behavioral Assessment

#### Open Field Test (OFT)

Manic-like behavior represented as increased locomotor activity can be reliably assessed in the OFT [8]. The apparatus is made of a wooden box with 40-cm-high white walls covered with water-resistant Formica. Its black floor (100 cm × 100 cm) is divided by white lines into 16 squares (25 cm × 25 cm). The test was performed in a sound isolated room under white light. Rats were gently placed in the central area of the box and the locomotor activity was video observed for 5 min. Then, the floor was wiped clean with 20% ethyl alcohol after each tested rat. Ambulation: (total number of square crossings during the entire test period) and rearing frequencies (total number of erect postures during the entire test period) as well as time spent in central area were recorded for each rat.

#### Forced Swim Test (FST)

FST was performed as previously described by Porsolt et al. [27]. Rats were individually placed in a Plexiglas cylinder (50 cm height × 20 cm diameter) containing tap water (25 ± 2 °C, 30 cm deep). The test was carried out for 2 days. On the first day (pretest session), rats were trained to swim for 10–15 min. On day 2 (testing session), same procedure as day 1 was performed for only 5 min. After each test, water was changed, the cylinder was washed, animals were dried and placed in their home cages under a heating lamp. The 5-min test was videotaped for recording the immobility and swimming time. Immobility defined as the animal floating without struggling and only making movements necessary to maintain its head above water, was used as an indicator of depression-like behavior [12].

### Tissue Processing

At the end of behavioral assessment, rats were euthanized under anaethesia by cervical dislocation and the PFC were rapidly dissected and stored at − 80 °C for further investigations. The PFC from each group were divided into three subsets; in the first set (n = 5), PFC were homogenized in ice-cold phosphate buffer saline (PBS, pH 7.4) for ELISA measurement. In the second set (n = 5), samples were homogenized in RNA lysis buffer for RT-PCR assessments. Meanwhile, in the last set (n = 5), PFC were homogenized in RIPA buffer (50 mM Tris HCl pH 8, 150 mM NaCl, 1% Triton X-100, 0.5% sodium deoxycholate and 0.1% SDS) provided with phosphatase inhibitor cocktail for Western blot analysis. Protein contents of the samples were estimated using Bradford assay [28].

During analysis, the investigators were blinded to sample identity. Sample coding and decoding were carried out by an independent experimenter.

### Biochemical Parameters

#### ELISA Assay for Adenosine, DA, Glutamate, BDNF, NF-κBp65, TNF- α and Il-27

Rat ELISA kits were used to estimate the PFC contents of adenosine, glutamate, NF-κBp65, Il-27 (MyBioSource, CA, USA); BDNF (Elabscience Biotechnology Co. Ltd.,Wuhan, China); DA and tumor necrosis factor-alpha (TNF-α) (Cusabio, Wuhan, China). All procedures were done according to the manufacturers’ protocols. The results are presented as ng/mg protein for DA and adenosine, pg/mg protein for BDNF, Il-27, NF-κBp65, TNF-α and nmol/mg protein for glutamate.

#### Quantitative Real-Time RT-PCR for β-Arrestin-2, NMDAR 2A, PKC-α and SNAP-25

Total RNA was extracted from PFC tissue using RNeasy Mini kit (Qiagen, Venlo, Netherlands) and the purity of obtained RNA was verified spectrophotometrically. The extracted RNA was then reverse-transcribed into complementary DNA using Reverse Transcription System (Promega, Leiden, Netherlands) according to the manufacturer’s procedure. Quantitative RT-PCR was performed to determine PFC gene expression of β-Arrestin-2, N-methyl-D-aspartate (NMDAR 2A), PKC-α and SNAP-25 using SYBR Green JumpStart Taq ReadyMix (Sigma-Aldrich, MO, USA) as described by the manufacturer. The sequences of primers are described in Table 1. The relative expression of target genes were obtained using the2^−ΔΔCT^ formula using β-actin as a housekeeping gene.

**Table 1:** Primer sequences used for RT-PCR

mRNA species	Primer sequence (5′‒3′)
β-Arrestin-2	F: AGCACCGCGCAGTACAAGT R: CACGCTTCTCTCGGTTGTCA
NMDAR 2A	F: TCCATTCTTCTGTCATCCTGC R: AAGACCGTCTCTCACTCTTGC
PKC-α	F: TTCCCAATCATCATAGCACA R: GAGATAGTTATCAACCGAGCAG
SNAP-25	F: GCAGGGTAACAAACGATGCC R: CTTCCCAGCATCTTTGTTGC
β-actin	F: TATCCTGGCCTCACTGTCCA R: AACGCAGCTCAGTAACAGTC

#### Western Blot Analysis for pS9-GSK-3β, pS473-Akt, pS552-β-catenin, pS41-GAP-43

In brief, PFC samples were homogenized in lysis buffer provided with complete protease inhibitor cocktail. After protein quantification using Bio-Rad Protein Assay Kit (Bio-Rad, CA, USA), an aliquot of 50 μg protein from each sample was separated by SDS polyacrylamide gel electrophoresis and transferred to a nitrocellulose membrane (Amersham Bioscience, NJ, USA) using a semidry transfer apparatus (Bio-Rad, Hercules, CA, USA). Precision Plus Protein All Blue standards (Bio- Rad, CA, USA) were used as molecular weight markers. The membranes were stained transiently with Ponceau S solution (Sigma–Aldrich, MO, USA) to verify protein transfer. Membranes were incubated with a blocking solution containing 5% nonfat dry milk in Tris-buffered saline with 0.05% Tween-20 (TBST) to prevent non-specific binding. Afterwards, blots were incubated overnight at 4 °C with primary antibodies against *p*S9-GSK-3β, *p*S473-Akt, *p*S552-β-catenin, *p*S41-GAP-43 and anti-β-actin polyclonal antibody (ThermoFisher Scientific, MA, USA) were diluted 1:1000 ratio. Next, they were washed and incubated with HRP-conjugated goat anti-rabbit IgG (Dianova, Hamburg, Germany). Finally, the band signal was developed using the enhanced chemiluminescence system (Amersham Biosciences, IL, USA) and the amount of protein was quantified by densitometric analysis using a scanning laser densitometer (GS-800 system, Bio-Rad, CA, USA) and normalized to that of loading controls namely GSK-3β, Akt, β-Catenin and GAP-43. Phosphorylation levels were evaluated by the ratio of phosphoprotein to total protein.

### Statistical Analysis

Results were tested for normality as well as homogeneity of variance using Kolmogorov–Smirnov and Bartlett’s tests, respectively. Data sets that met the assumptions for parametric analysis were analyzed using one-way analysis of variance (ANOVA) followed by the Tukey’s Multiple Comparison’s test and were expressed as mean ± SD. Statistical analysis was carried out using GraphPad Prism software package, version 7 (GraphPad software Inc., CA, USA). The level of significance was set to *p* <  0.05 for all statistical tests.

## Results

Since both SCH58261 and Li treated groups did not show any significant difference as compared to the control group, therefore, all comparisons were conducted against the control group only.

### Effect of SCH58261 and Li on MPH-induced Alterations in Locomotor Activity and Depressive Behavior

**Fig. 2:**
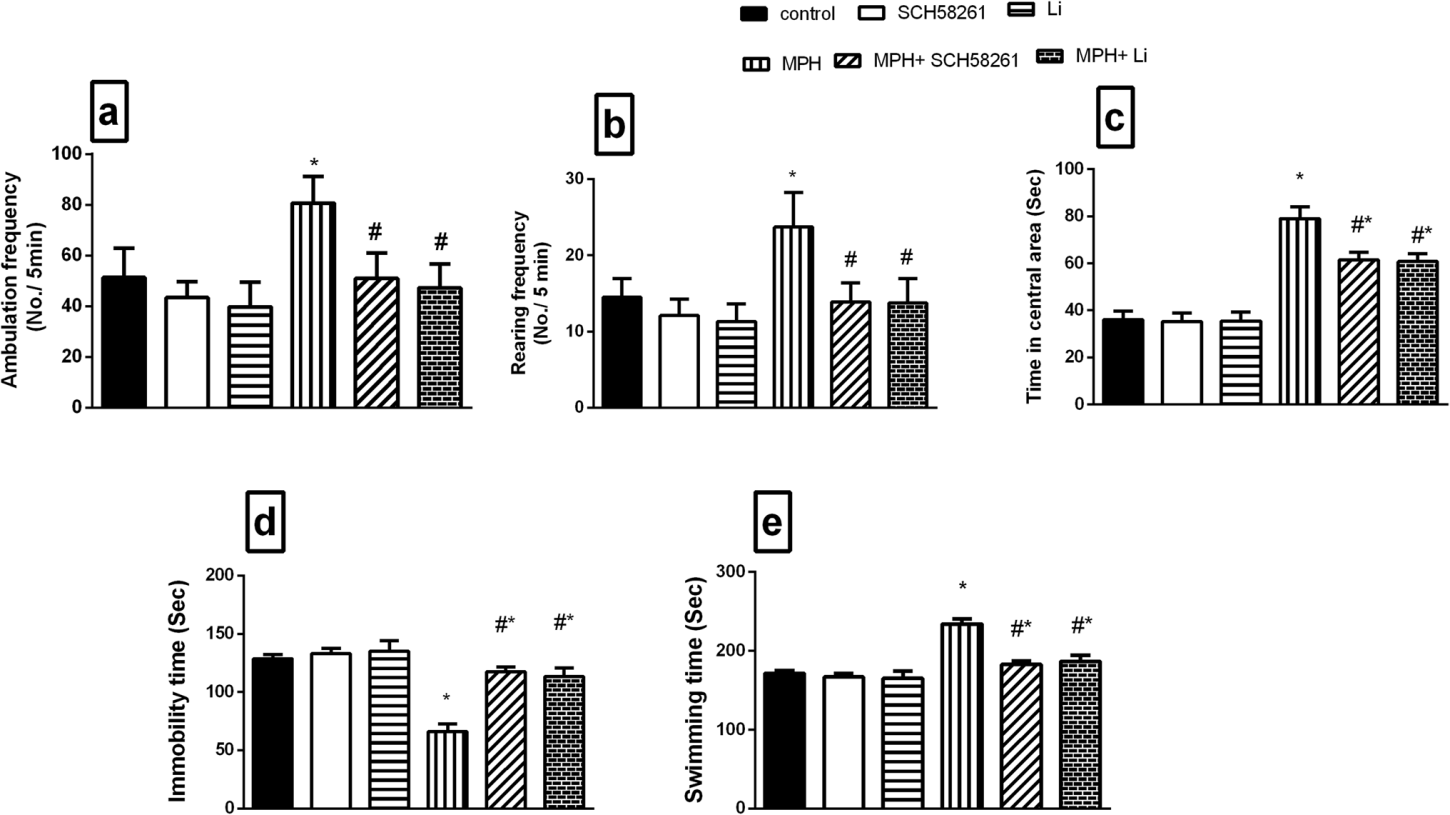
**Effect of SCH58261 and Li on MPH-induced alterations in rats’ behavior in the open field (OFT); (a) ambulation frequency, (b) rearing frequency, (c) time in central area and in the forced swim (FST) test; (d) immobility time and (e) swimming time.** Values are expressed as the mean ± S.D. of 15 rats/group. Statistical analysis was performed using one-way ANOVA followed by Tukey’s Multiple Comparisons test. Values are statistically significant at *p* < 0.05. * versus the control group, # versus the MPH group. MPH: methylphenidate; Li: lithium

In the open field test, intraperitoneal injection of MPH resulted in a significant elevation in the ambulation (Fig. 2a) and rearing (Fig. 2b) frequencies as well as time spent in central area (Fig. 2c) as compared to the control group indicating hyperactivity, impulsivity and risk-taking behavior. Additionally, in the forced swim test, MPH-treated rats exhibited mania-like behavior reflected as a significant prolongation in the swimming time (Fig. 2e) together with a reduction in the immobility time (Fig. 2d) compared to the control group. Interestingly, treatment with either SCH58261 or Li-normalized ambulation, rearing frequencies and reduced time spent in central area and significantly ameliorated forced swim parameters as compared to the diseased group.

### Effect of SCH58261 and Li on MPH-induced Alterations in Neurotransmitters as well as the Gene Expression of NMDAR 2A in the PFC

**Fig. 3:**
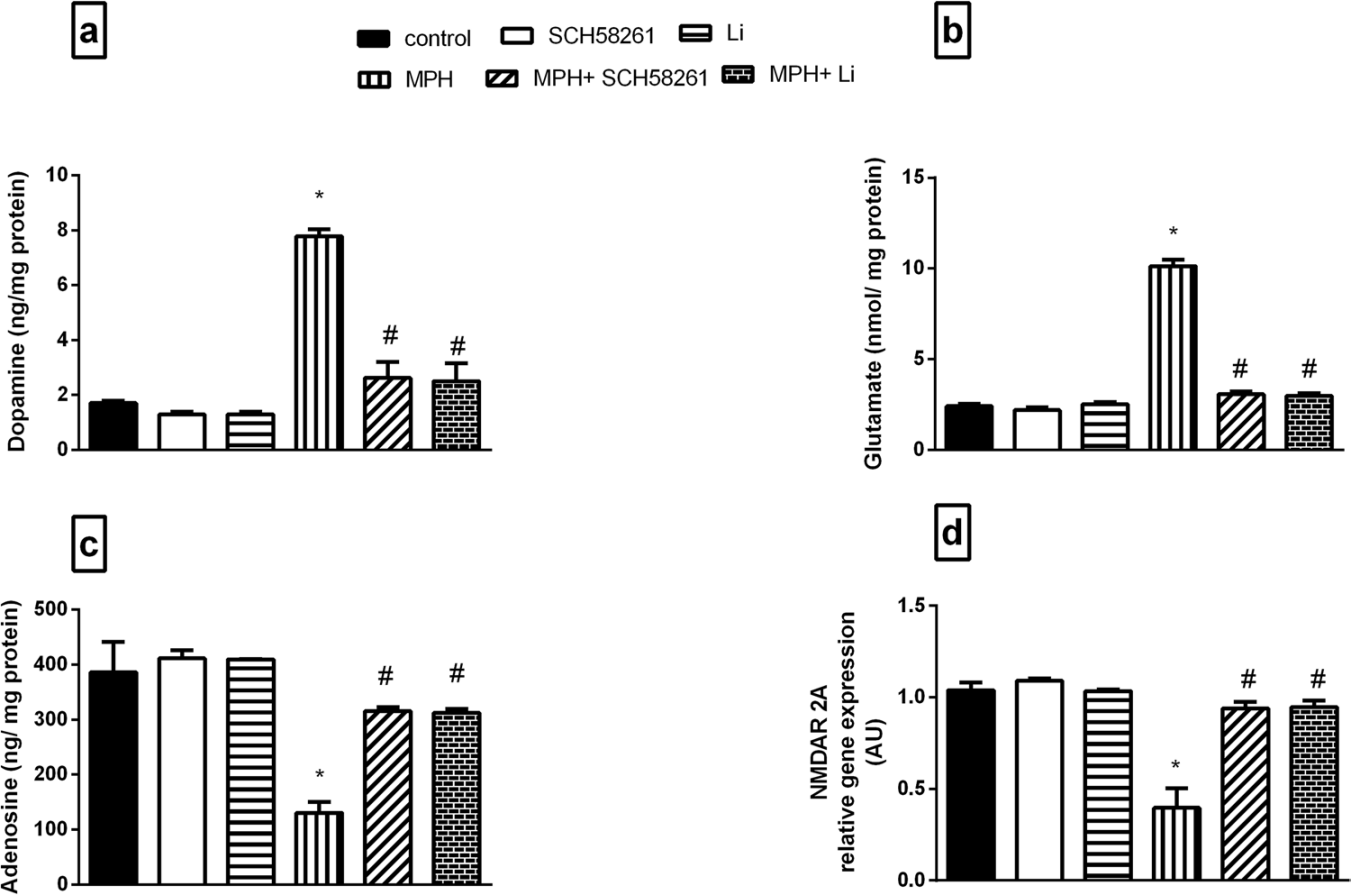
**Effect of SCH58261 and Li on MPH-induced alterations in (a) dopamine, (b) glutamate, (c) adenosine and (d) NMDAR 2A.** Values are expressed as the mean ± S.D. (n = 5). Statistical analysis was performed using one-way ANOVA followed by Tukey’s Multiple Comparison’s test. Values are statistically significant at *p* < 0.05. * versus the NC group, # versus the MPH group. MPH: methylphenidate; Li: lithium; NMDAR 2A: NMDA receptor subunit 2A

MPH-injected animals demonstrated a pronounced escalation in the PFC DA (Fig. 3a) and glutamate (Fig. 3b) contents as related to the control animals. Such increments were hampered by both SCH58261 and Li administration causing a normalization in their contents. In contrast, MPH regimen significantly mitigated adenosine content as well as the mRNA expression of NMDAR 2A as related to the control group, an effect that was normalized by either SCH58261 or Li treatments (Fig. 3c and d).

### Effect of SCH58261 and Li on MPH-induced Alterations in PKC-α Signaling as well as β-Arrestin-2/Akt/GSK/β-catenin Pathway

**Fig. 4:**
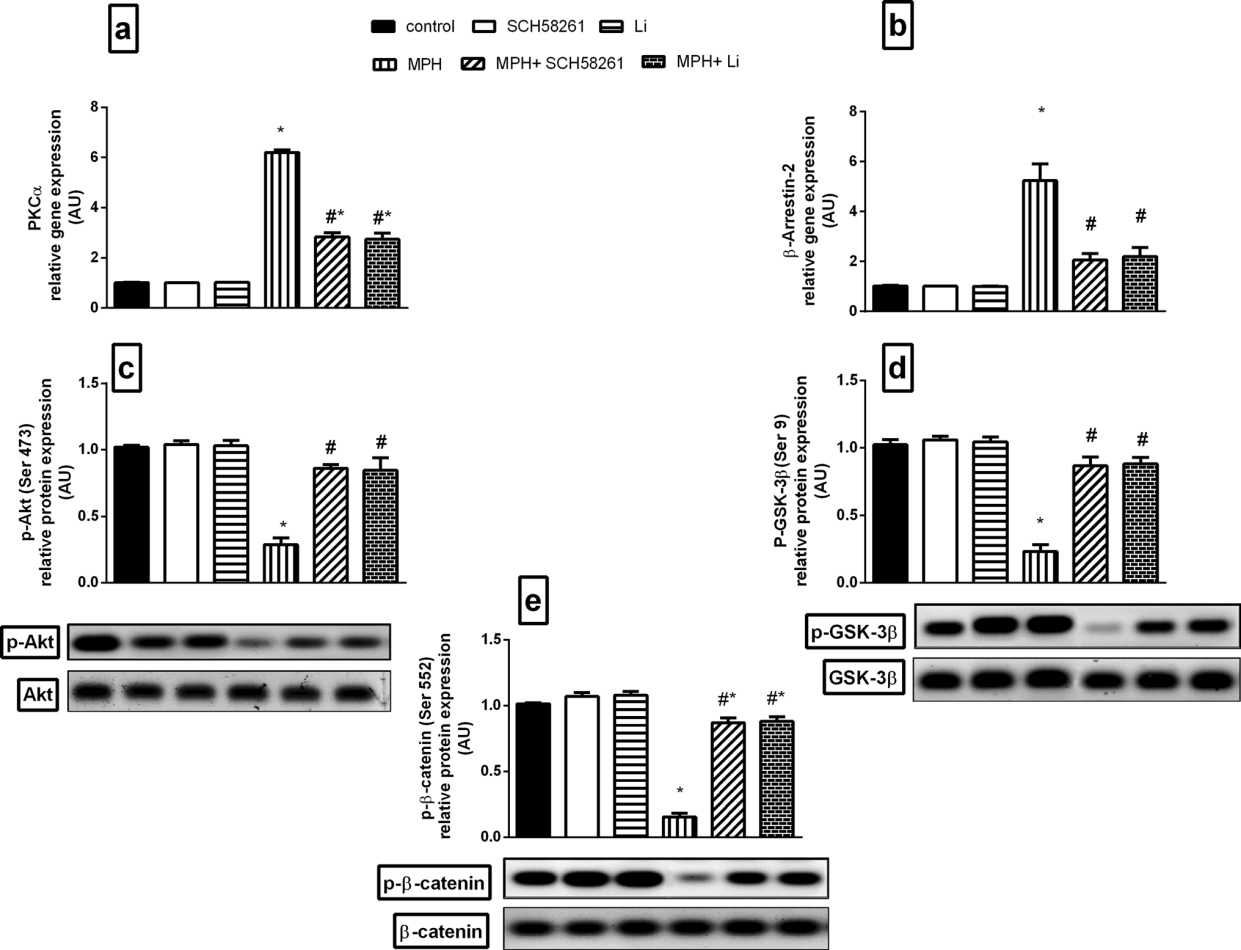
**Effect SCH58261 and Li on MPH-induced alterations in PKC-α, β-Arrestin-2, pS473-Akt, pS9-GSK-3β and pS552-β-catenin in rats. (a) PKC-α, (b) β-Arrestin-2, (c) pS473-Akt, (d) pS9-GSK-3β and (e) pS552-β-catenin.** Values are expressed as the mean ± SD (n = 5). Statistical analysis was performed using one-way ANOVA followed by Tukey’s Multiple Comparison. Values are statistically significant at *p* < 0.05. * versus the control group, # versus the MPH group. MPH: methylphenidate; Li: lithium; PKC-α: protein kinase-c alfa; p-S473-Akt: p-S473 protein kinase B; p-GSK-3β: p-S9 glycogen synthase kinase-3 beta

Repeated MPH administration produced a substantial elevation in PKC-α (Fig. 4a) and β-Arrestin-2 (Fig. 4b) mRNA expressions in the PFC as compared to the control group. These effects were significantly reversed by both SCH58261 and Li treatments as compared to MPH rats. Conversely, MPH produced a pronounced reduction in *p*S473-Akt (Fig. 4c) with subsequent reduction in *p*S9-GSK-3β (Fig. 4d) and *p*S552-β-catenin (Fig. 4e) as compared to the control group. However, treatment with either SCH58261 or Li upregulated the phosphorylation of *p*S473-Akt, β-catenin and normalized GSK-3β as compared to MPH rats.

### Effect of SCH58261 and Li on MPH-induced Alterations in Synaptic Plasticity Markers

**Fig. 5:**
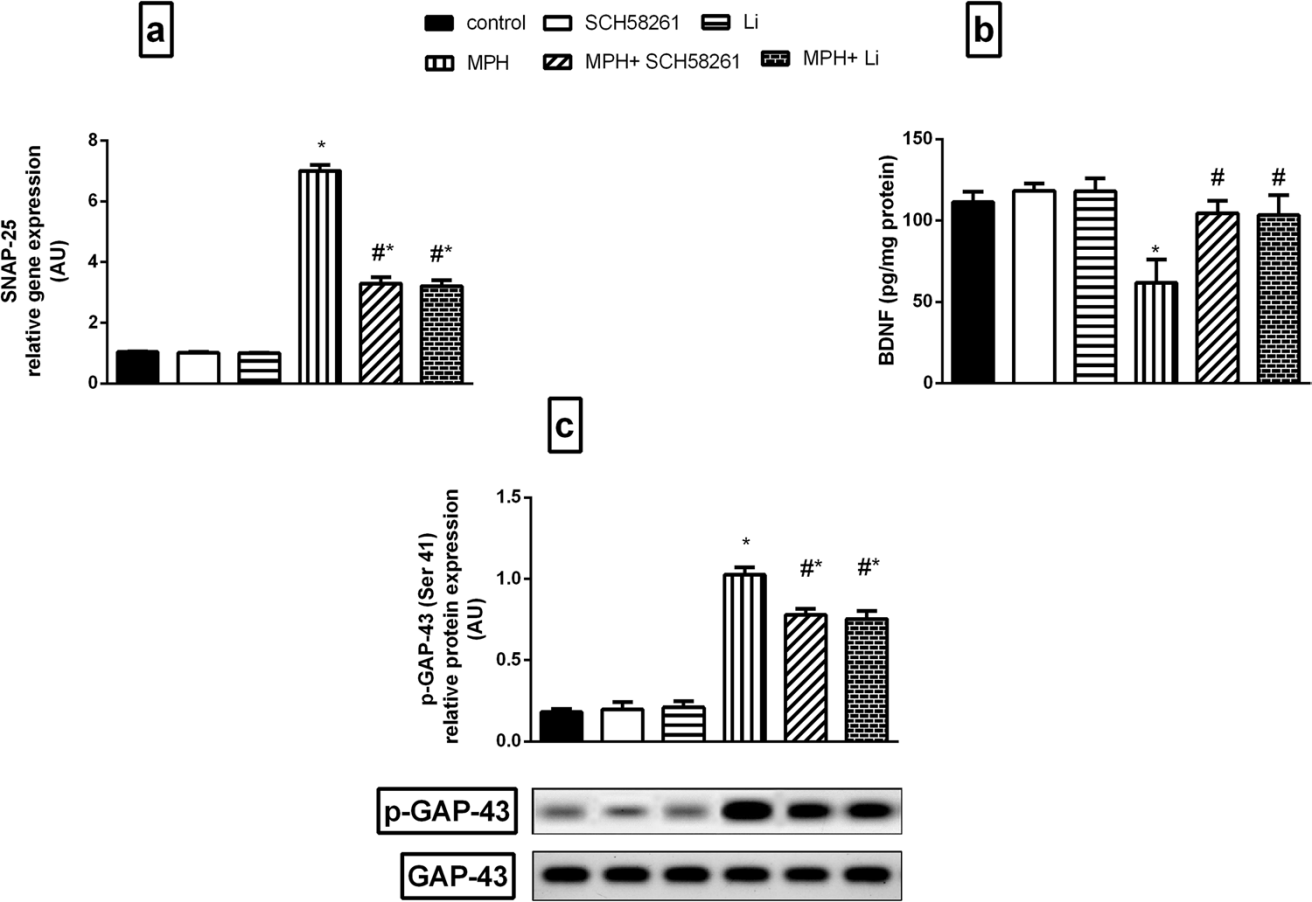
**Effect SCH58261 and Li on MPH-induced alterations in SNAP-25, BDNF and pS41-GAP-43 in rats. (a) SNAP-25, (b) BDNF and (c) pS41-GAP-43.** Values are expressed as the mean ± SD (n = 5). Statistical analysis was performed using one-way ANOVA followed by Tukey’s Multiple Comparison. Values are statistically significant at *p* < 0.05. * versus the control group, # versus the MPH group. MPH: methylphenidate; Li: lithium; SNAP-25: synaptosomal-associated protein-25; pGAP-43: p-S41 Growth Associated Protein 43; BDNF: brain derived neurotrophic factor

Compared with the control group, injection of MPH significantly increased mRNA expression of SNAP-25 (Fig. 5a) as well as *p*S41-GAP-43 (Fig. 5c) protein expression together with a reduction in BDNF content (Fig. 5b) as compared to their control counterparts. On the contrary, treatment with either SCH58261 or Li significantly reduced SNAP-25 and *p*S41-GAP-43 expression and elevated BDNF content to normal values as compared to MPH-treated group.

### Effect of SCH58261 and Li on MPH-induced Inflammatory Changes

**Fig. 6:**
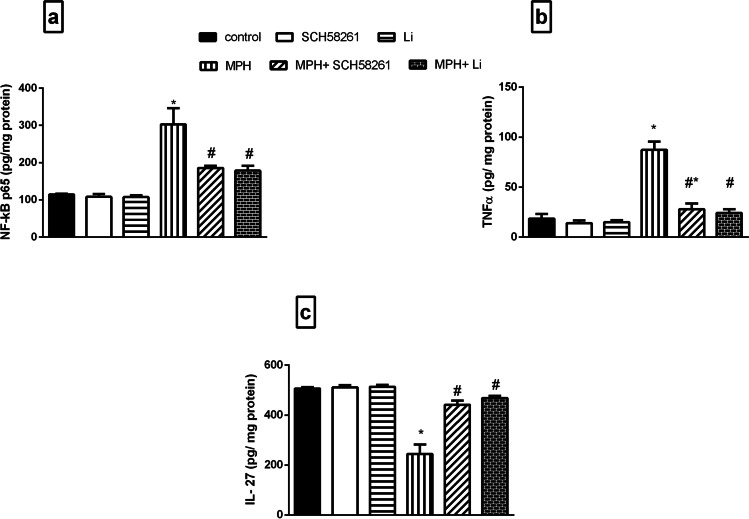
**Effect SCH 58,261 and Li on MPH-induced alterations in PFC NF-κBp65, TNF-α and IL-27 in rats. (a) NF-κBp65, (b) TNF-α and (c) IL-27.** Values are expressed as the mean ± SD (n = 5). Statistical analysis was performed using one-way ANOVA followed by Tukey’s Multiple Comparison. Values are statistically significant at *p* < 0.05. * versus the control group, # versus the MPH group. group MPH: methylphenidate; Li: lithium; NF-κBp65: nuclear factor kappa-light-chain-enhancer of activated B cells; TNF-α: tumor necrosis factor alpha; IL-27: interleukin 27

Figure 6 demonstrates that MPH significantly increased NF-κBp65 (Fig. 6a) and TNF-α contents (Fig. 6b), while profoundly diminished IL-27 content (Fig. 6c) as compared to the control group. However, treatment with either SCH58261 or Li ameliorated such inflammatory events through reducing NF-κBp65 and TNF-α with a significant increment in IL-27 content as compared to MPH group.

## Discussion

Away from the traditional theories in neuropsychiatric disorders, and owing to the multiple side effects of the standard treatments, ongoing research for new therapeutic targets is no more an option. In manic disorders, neuromodulators including adenosine have been now focused on. Nonetheless, the role of adenosine receptor signaling in such disorder remains elusive. In the current study, a selective adenosine A_2A_R antagonist (SCH58261) was used as a testing tool to study the effect of adenosine A_2A_R blockade in comparison to lithium as the most effective anti-manic drug. Notably, adenosine A_2A_R inhibition resulted in a feedback elevation of prefrontal cortical adenosine together with the reduction in the cardinal neurotransmitter DA involved in mania. Meanwhile, SCH58261 suppressed glutamate with an upregulated protein expression of NMDAR 2A subunit to restore NMDA receptor function, which is a major player in manic disorder pathophysiology. A_2A_R antagonism also downregulated PKC-α and β-Arrestin-2 gene expressions to activate/phosphorylate Akt at Ser 473 that consequently counteract GSK-3β deleterious effects. In further extension to the benefits of A_2A_R deactivation in MPH-induced mania, it was shown herein that SCH58261 regulated synaptic plasticity markers through increasing BDNF along with downregulating GAP-43, SNAP-25 and ameliorating neuro-inflammation through reducing NF-κBp65 and TNF-α along with elevating IL-27. The above-mentioned effects induced by adenosine A_2A_R blockade were all reflected as improved behavioral/biochemical profile and were comparable to the standard drug lithium to announce the adenosine A_2A_R as a novel therapeutic target and open a new avenue for mania management.

In the CNS, adenosine was proposed to control the release of other neurotransmitters [5]. Adenosine level has been found to be declined following a variety of brain insults including schizophrenia and bipolar disease [5, 29]. According to Boison et al. [5], purinergic system malfunction accounts for the imbalance between dopaminergic and glutamatergic neurotransmission as characteristic hallmarks of schizophrenia. In the current study, the role of such triad of contributors namely, adenosine, DA and glutamate in the psychostimulant effects of MPH has been addressed. MPH suppresses the mitochondrial respiratory chain (complex I, II, III, and IV) [30] thus reducing adenosine triphosphate (ATP) production with a subsequent reduction of adenosine levels as observed herein in agreement with another report [31]. Notably, Adenosine A_2A_R is selectively upregulated in glutamatergic nerve terminals as well as astrocytes thus triggering schizophrenia-like symptoms [32]. The notion of dysregulated dopaminergic neurotransmission was also established in the pathophysiology of various diseases such as schizophrenia and bipolar disorder [33]. Notably, the locomotor hyperactivity induced by MPH has been reported [11] which is associated by a robust increment in DA release in the PFC [34]. This upsurge is attributed not only to MPH ability to increase DA release but also to inhibition of dopamine transporter (DAT) [35] with subsequent decrease in DA uptake an effect mediated by PKC-α phosphorylation of N-terminal of DAT [9, 36]. In the current study, MPH administration induced a significant reduction in adenosine levels along with increased DA and glutamate levels accounting for MPH-induced mania like symptoms as observed by increased locomotor activity, impulsivity and risk-taking behavior in the open field and decreased immobility time in the forced swim test. These events were reversed following administration of the A_2A_R antagonist (SCH58261). Despite the divergence of reports on the impact of A_2A_R on hyperlocomotion, anxiety-like behavior and depressive effects, which is mostly attributable to the differential contribution of A_2A_R in different brain regions [37], herein, A_2A_R blockade in the PFC resulted in reduced hyperlocomotion and risk taking behavior in the open field which is possibly due to the synergestic nature of the selective A_2A_/D_2_ interaction in this region of the brain. Indeed, it was evidenced that contrary to other brain regions, the synergistic A_2A_R/D_2_R interaction in the PFC, accounts for most psychotic and mania like symptoms. Further, in the FST, high level of swimming is consistent with manic behavior [38, 39], where repeated administration of MPH in this study induced locomotor hyperactivity which might be responsible for the decreased immobility observed in FST in line with a previous study [40]. Indeed, A_2A_R blockade reduced the behavioral effects of DA possibly through inhibiting D_2_ receptors proclaiming the potential of A_2A_R as a major player in regulating the pathophysiology of manic disorders which still remains to be explored. In support, a study by Kaster et al. [41] revealed that SCH58261 normalized immobility time in FST in a model of chronic stress.

Studies have shown that A_2A_R controls the D_2_R effect on glutamatergic transmission, where D_2_R reduces glutamate uptake in the PFC leading to an increased availability of glutamate in the synaptic cleft [2]. In this study, MPH administration resulted in increased both glutamate and DA levels in the PFC which could be partly due to glutamate uptake reduction following A_2A_/D_2_R activation. Effects that were reversed following the A_2A_R antagonist (SCH58261) administration. Another plausible explanation for the increased glutamate is the NMDA receptor hypofunction observed in the current study which might have produced a paradoxical increment in glutamate levels. Notably, a defective NMDAR 2A subunit in the PFC of MPH-treated rats is the main cause of NMDA hypofunction in support with other studies indicating NMDAR hypofunction in schizophrenia and other psychiatric illnesses [42]. There is a piece of evidence that under pathological conditions, A_2A_R signaling is exaggerated leading to early cognitive and synaptic dysfunction as well as neurodegeneration [43] where A_2A_R dysfunction may even precede NMDAR dysfunction [44]. Hence, blocking adenosine A_2A_R might have rebalanced NMDAR hypofunction either directly through upregulating NMDAR 2A subunit or indirectly through inhibiting D_2_ receptors and reducing glutamate release thus ameliorating the deleterious excitotoxic effects.

Motor hyperactivity and risk-taking behavior have been also linked to overactive PKC-α signaling in the PFC [45]. The present investigation revealed that administration of MPH was associated with enhanced PKC-α activity in line with a previous study [46]. The activated A_2A_R following MPH administration enhanced PKC-α activity in line with other studies showing that A_2A_R activation increases PKC-α [47]. Worthy of note, A_2A_R acts through either cAMP/PKA or DAG/PKC-α where elevated glutamate levels are known to shift the signaling towards PKC-α pathway inducing damaging effects as observed herein. Further, adenosine A_2A_R facilitation of synaptic transmission involves PKC-α rather than PKA activation [48]. The A_2A_-dependent PKC-α effects also play a pivotal role in regulating adenosine nucleoside transporters and modulating adenosine release and uptake [49]. Those mentioned effects of A_2A_R on neuromodulation through direct coupling to PKC-α have pointed to the importance of exploring the A_2A_-PKC-α–dependent effects in MPH-induced mania. In the present study, injection of the A_2A_R antagonist, SCH58261 markedly suppressed PKC-α activity in parallel with another study [50]. In the same context, other studies reported that the SCH58261-induced neuroprotection in brain ischemic models could be related to PKC-α inhibition [51]**.** Moreover, treatment with the anti-manic drug Li successfully abolished PKC-α activity in line with another study [14], an effect related to the inhibition of its translocation from cytosol to cell membrane which is in harmony with our study.

Indeed, multiple pathways are activated downstream from PKC-α. One major signaling is the Akt/GSK-3β/β-catenin cascade which influence many critical cellular functions such as gene expression, neural plasticity, cell structure and cell survival [52]**.** Dysregulated Akt/GSK-3β/β-catenin pathway is directly associated with the most prevalent neuropsychiatric disorders such as schizophrenia, anxiety, depression, and bipolar disorder [52]. In this work, Akt is dephosphorylated/inactivated with concomitant activation of its downstream target GSK-3β leading to a plethora of damaging events. Such inhibition of Akt could be explained by two mechanisms; (1) Elevated PKC-α activates Protein phosphatase 2A (PP2A) which promotes dephosphorylation of Akt at Ser 473 as observed herein in accordance with other reports [53], (2) Hyperdopaminergic status through binding of DA to D_2_ receptor is associated with the recruitment of β-Arrestin-2 and the formation of Akt/β-Arrestin-2/PP2A complex. In this way, Akt is dephosphorylated leading to inhibition of its activity [54]**.** A growing evidence points to GSK-3β as a pro apoptotic molecule and its dysfunction is correlated to the pathophysiology of mood disorders [55]. Moreover, GSK-3β inhibition has attracted widespread attention as one of the critical therapeutic targets where its inhibition could afford anti-manic effect [56]. Notably, the actions of GSK-3β are negatively regulated by its phosphorylation at Ser 9 [55] which is in line with our report that shows increased phosphorylation of GSK-3β (Ser 9) upon SCH58261 administration thus reducing its deleterious effects. In harmony with our previous findings, a study by Schmitz et al. [57] has reported a diminished Akt activity in response to MPH treatment, which could be linked to increased DA level. These former events are all synchronized herein with administration of the A_2A_R antagonist which significantly decreased β-Arrestin-2 along with elevating *p*S473-Akt and *p*GSK-3β (Ser 9) with concomitant inhibition of GSK-3β deleterious effects. Another add-on benefit for A_2A_R antagonism is the elevation of BDNF as an essential neurotrophin. Indeed, BDNF is another negative regulator of GSK-3β activity where, such inhibitory effect is achieved by binding of BDNF to tyrosine receptor kinase (TrkB) with subsequent activation of PI3K/Akt pathway and hence, inhibition of GSK-3β by its phosphorylation at Ser 9 [58]. Extensive evidence implicated reduction in BDNF level in patients suffering from bipolar during the manic phase [59]. Further, previous studies reported decreased BDNF levels following MPH which confirmed our results [25]**.** There is ample evidence of an interaction between A_2A_R and BDNF in different brain regions**.** Interestingly, A_2A_R blockade in the hippocampus was found to increase BDNF signaling [60–62] while genetic elimination of A_2A_R in the forebrain triggers BDNF decrement in the cortex [63]. However, under certain conditions, BDNF may trigger microglial proliferation and exacerbate neuro-inflammation [64, 65]. In the present investigation, the anti-manic effect of SCH58261 was manifested by the prominent augmentation of BDNF level. This favorable effect is strongly correlated with its ability to activate Akt along with reversing the MPH-induced elevation of GSK-3β activity. Consistent with our data, a previous study showed that lithium increases BDNF level through enhancing Akt-mediated inhibition of GSK-3β [24].

Notably, active GSK-3β is involved in manipulating vital signaling molecules including β-catenin among others. This is a key molecule for cell proliferation, differentiation, neuronal survival, neuro-inflammation, and synaptic plasticity [17]. When GSK-3β is activated, it promotes β-catenin degradation in the absence of Wnt [66]. In the same context, the inhibited Akt as observed herein resulted in a marked reduction of β-catenin phosphorylation/activation at Ser 552 thereby reducing several genes expression such as IL-27 and NMDAR 2A in agreement with another report [66, 67]. Administration of the A_2A_ antagonist significantly reversed such effects through negatively regulating GSK-3β together with phosphorylating β-catenin at ser 552 resulting in its activation. Moreover, activation of β-catenin in this way might have rebalanced the NMDA receptor hypofunction through increasing NMDAR 2A expression as shown in this study in line with another report confirming the link between β-catenin and NMDAR 2A expression in the PFC [66]. In agreement with our results, a previous study revealed the efficiency of Li in preventing β-catenin degradation and enhancing NMDAR 2A subunit expression in the PFC, an effect that is strongly correlated to its ability to suppress GSK-3β activity [66].

Interestingly, the neuromodulin GAP-43 is a direct participant in axonal growth regulation and formation of neural network [68]. This protein is one of the main substrates for PKC-α in the brain that plays a role in long-term potentiation. Studies have shown that PKC-α phosphorylates GAP-43 at ser 41 increasing its expression [19]. Moreover, such phosphorylation facilitates binding of GAP-43 to cytoskeletal proteins and hence membrane formation [69]. Notably, genetic deletion of GAP-43 gene resulted in early death in the postnatal period in mice [69]. On the other hand, levels of GAP-43 were upregulated in other brain disorders including stroke [70], schizophrenia [20] and bipolar disorder [19]. In agreement, MPH administration in this study elevated GAP-43 phosphorylation at ser 41 which is attributed to overactive PKC-α. This finding goes inline with other studies indicating upregulated GAP-43 after treatment with MPH/amphetamine psychostimulants [19, 71]**.** Indeed, A_2A_R blockade by SCH58261 reduced the effect of MPH on this protein phosphorylation reinforcing the importance of modulating PKC-α in amelioration of mania-like symptoms associated with psychostimulants. Such effect of A_2A_R antagonism is comparable to that produced by the anti-manic drug lithium in line with another report [14].

Another effector as a downstream from PKC-α is SNAP-25 [14]. This protein receptor is required for the vesicular transport of proteins and accounts for membrane fusion by forming a complex that fuses the synaptic vesicle to plasma membranes [72]. Upon its activation, PKC-α phosphorylates SNAP-25 at Ser 187 facilitating neurotransmitter release [73]. Indeed, a positive correlation was verified between mania-like behavior and SNAP-25 phosphorylation. In the present study, MPH-elevated SNAP-25 expression in the PFC in consistence with a former report by Kim et al. [74]. Events that were all reversed following both SCH58261 and lithium administration possibly due to hindering PKC-α signal. In support to our findings, A_2A_R blockade using SCH58261 was proven to be effective in maintaining synaptic integrity and preventing synaptotoxicity caused by different insults such as Alzheimer’s disease [75, 76], chronic stress [41, 77], and convulsion [78].

Studies have indicated that manic episodes are associated with a pro-inflammatory state [79]. Furthermore, regulating the expression of inflammatory cytokines is considered as one of the GSK-3β/β-catenin downstream signaling molecules that is responsible for maintaining the balance between the pro-inflammatory and the anti-inflammatory cytokines [80]. Indeed, reduction of the active β-catenin as demonstrated herein diminishes the production of anti-inflammatory cytokines as IL-27 thus shifting the balance towards pro-inflammatory cytokine production [67]. Moreover, GSK-3β is established to promote neuro-inflammation and cytokine production via regulating NF-κBp65 [81]**.** It has been established that administration of MPH triggers the inflammatory response [34]. In support, the present work confirmed that MPH treatment aggravated the inflammatory status as evidenced by a significant increment in NF-κBp65 and TNF-α levels as well as reduction of the anti-inflammatory cytokine IL-27. Studies have shown that A_2A_R controls neuro-inflammation, characterized by the appearance of activated inflammatory microglial cells and increase in the levels of inflammatory mediators [82]. Noteworthy, microglial cells, which are shown to express A_2A_R, are the primary source of TNF-α in the brain [83, 84]. Previous studies have demonstrated the anti-inflammatory effects of SCH58261 in models of brain ischemia [23], spinal cord injury [85] and multiple sclerosis [86]. Further, a previous study from our lab has shown that SCH58261 reduced microglial activation and neuro-inflammation [23]. Indeed, SCH58261-treated rats in the current study exhibited a significant diminution in NF-κBp65 and TNF-α levels along with an upsurge of IL-27 level. These observed effects were comparable to those obtained by treatment with lithium as an established mood stabilizer in line with a previous study [87].

## Conclusion

**Fig. 7:**
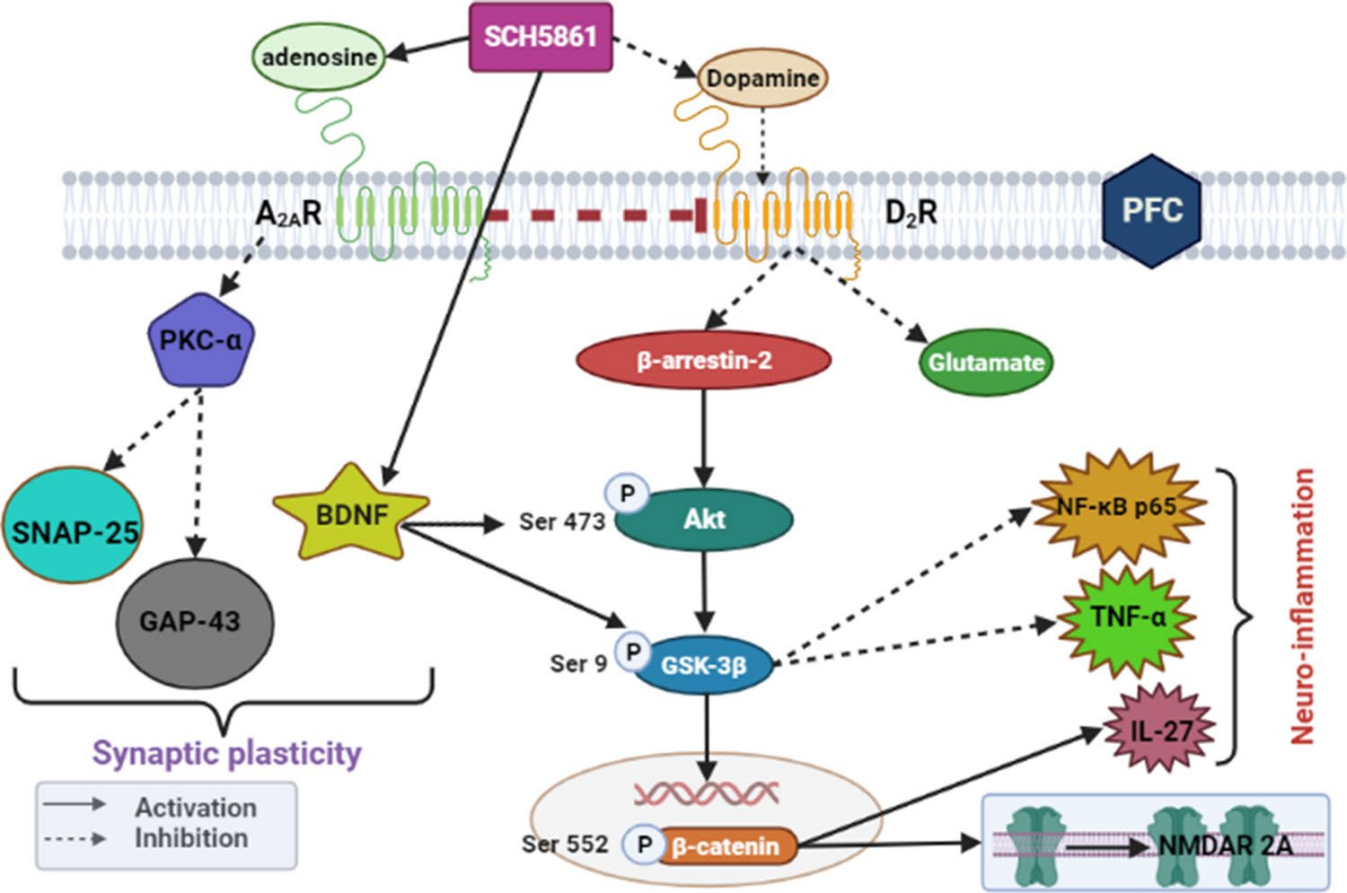
**Graphical abstract: Graphical illustration for the proposed mechanisms of the anti-manic potential of SCH58261.** BDNF: brain derived neurotrophic factor;; *pS9-*GSK-3β: *p-*S9 glycogen synthase kinase-3 beta; IL-27: interleukin 27; *pS41-*GAP-43: *p*-S41 Growth Associated Protein 43; NMDAR 2A: NMDA receptor subunit 2A; NF-κBp65: nuclear factor kappa-light-chain-enhancer of activated B cells; PKC-α: protein kinase-c alfa; *p*S473-Akt: *p*-S473 protein kinase B; TNF-α: tumor necrosis factor alpha;; SNAP-25: synaptosomal-associated protein-25

The current study introduces adenosine A_2A_R blockade as a promising therapeutic option in ameliorating MPH-induced hyperactivity and mania-like symptoms. These effects could be linked to the ability of SCH58261 (A_2A_R antagonist) to suppress PKC-α activity in the PFC and promote Akt/GSK-3β/β-catenin signaling pathway (Fig. 7) thus producing a robust protective effect resembling that of the well-established anti-manic drug (lithium).

## Data Availability

The datasets generated and/or analyzed during the current study are available from the corresponding author upon reasonable request.
